# A Case of Rapidly Progressive Subglottic Hemangioma in an Infant: Early Propranolol Therapy Prevented Tracheostomy

**DOI:** 10.7759/cureus.93798

**Published:** 2025-10-03

**Authors:** Genki Iwai, Takeshi Takahashi, Hironori Baba, Ko Matsui, Arata Horii

**Affiliations:** 1 Otolaryngology - Head and Neck Surgery, Niigata University Graduate School of Medical and Dental Sciences, Niigata, JPN; 2 Otolaryngology - Head and Neck Surgery, Niigata University, Niigata, JPN; 3 Pediatrics, Niigata University Graduate School of Medical and Dental Sciences, Niigata, JPN; 4 Otolaryngology - Head and Neck Surgery, Niigata University Hospital, Niigata, JPN

**Keywords:** airway obstruction, hemangioma, infant newborn, propranolol, subglottis

## Abstract

Subglottic hemangioma is a rare but potentially life-threatening cause of neonatal airway obstruction. We report the case of a preterm infant who developed rapidly progressive biphasic stridor at 36 days of life. Flexible laryngoscopy revealed a subglottic mass, and contrast-enhanced computed tomography demonstrated a well-circumscribed, enhancing lesion causing >70% airway narrowing. Following careful airway planning, the patient was intubated safely and treated with oral propranolol, resulting in marked regression within 10 days and successful extubation without tracheostomy. The infant remained symptom-free, with no recurrence at 24 months. This case highlights the importance of early recognition of biphasic stridor, prompt laryngoscopic evaluation, and timely propranolol initiation, which may avert invasive surgical interventions.

## Introduction

Infantile hemangiomas, the most common benign vascular tumors in children, are characterized by rapid vascular growth during the first months of life [1]. Although they most frequently occur in the head and neck region, airway involvement is rare (~1.8% of cases) [2], with subglottic hemangiomas representing the most clinically significant and potentially life-threatening form. Most cases progress gradually, but some deteriorate rapidly and require urgent airway intervention [3-5]. Overlapping symptoms with upper respiratory infections can lead to misdiagnosis and delays in treatment. Contrast-enhanced computed tomography (CT) is useful for assessing the extent of disease, while laryngoscopy provides essential information for airway planning. We describe a preterm infant with a rapidly progressive subglottic hemangioma who was successfully managed with early propranolol therapy, avoiding tracheostomy; sedated flexible laryngoscopy confirmed a thick, nonfriable capsule with a low risk of contact-induced bleeding, permitting safe, controlled endotracheal intubation.

## Case presentation

**Figure 1:**
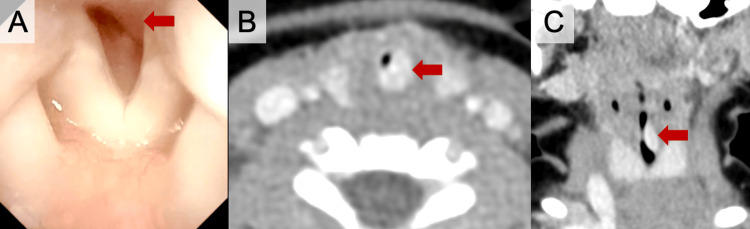
Pretreatment Laryngoscopic and CT Findings of Subglottic Hemangioma Pretreatment flexible laryngoscopy shows a smooth, compressible subglottic lesion (A), while axial (B) and coronal (C) contrast-enhanced CT demonstrate a strongly enhancing posterior subglottic mass (red arrow) causing more than 70% airway narrowing. CT, computed tomography

A 41-day-old female infant, born prematurely at 31 weeks and 5 days of gestation (corrected age: 35 weeks), had no complications at birth and no abnormalities were noted at the routine health checkup on day 30 of life. Stridor first appeared on day 36 and progressed rapidly over the following days. On presentation, her body temperature was 37.3°C, heart rate 122 beats/min, and oxygen saturation was 100% on 1 L/min of nasal oxygen. No wheezes, crackles, or cutaneous hemangiomas were noted. Suprasternal and intercostal retractions were prominent during crying but absent at rest. Arterial blood gas analysis revealed pH 7.181, PaCO₂ 81.6 mmHg, PaO₂ 43 mmHg, and HCO₃⁻ 30.5 mmol/L, indicating primary respiratory acidosis with partial metabolic compensation. Without evidence of lower airway or lung parenchymal disease, upper airway obstruction was suspected. The differential diagnosis at this stage included common congenital causes of neonatal stridor, such as laryngomalacia, vocal cord paralysis, subglottic cysts, and other structural anomalies, in addition to acquired conditions such as infection or inflammatory edema. Laryngomalacia was considered less likely because the stridor was biphasic rather than inspiratory only. To further characterize the lesion and confirm the site of obstruction, flexible laryngoscopy was performed, revealing a smooth, reddish, and non-ulcerated subglottic submucosal mass (Figure 1A).

Contrast-enhanced CT demonstrated a well-circumscribed, intensely enhancing mass in the left posterior subglottis, compressing the airway by more than 70% (Figures 1B-1C). The lesion measured approximately 10 mm in the vertical dimension, without proximal tracheal extension. These findings were consistent with subglottic hemangioma. Bleeding from the tumor due to traumatic intubation and the potential need for tracheostomy in case of prolonged airway support were concerns for securing the airway. Repeated flexible laryngoscopy under sedation confirmed a soft, thickly encapsulated lesion that would have a low risk of bleeding (Video 1).

**Video 1:** Laryngoscopic Views Before and After Propranolol Therapy Flexible laryngoscopic views before and 10 days after treatment.

**Figure 2:**
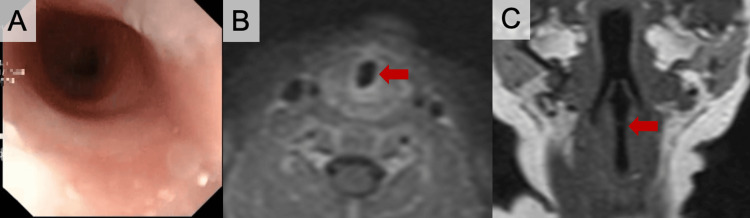
Posttreatment Laryngoscopic and MRI Findings Showing Lesion Regression Follow-up laryngoscopy performed immediately after extubation (A) and MRI (B, C) reveal marked regression of the subglottic hemangioma with improvement in airway patency. Red arrows indicate the widened airway lumen. MRI, magnetic resonance imaging

Endotracheal intubation was performed, and oral propranolol was initiated at 1 mg/kg/day, then titrated to 2 mg/kg/day by day 4. Continuous monitoring of heart rate, blood pressure, and blood glucose was undertaken during the initiation of propranolol. Transient hypotension was observed but resolved promptly after adjustment of the sedative medication. The infant was successfully extubated on day 10 without requiring tracheostomy. Follow-up laryngoscopy and magnetic resonance imaging (MRI) showed marked regression and airway improvement (Figure 2).

Propranolol was continued until 14 months of age. At the most recent follow-up at 24 months, no recurrence or residual respiratory symptoms were noted, and growth and development were appropriate for age (Table 1).

**Table 1:** Key Investigations, Airway Assessment, and Propranolol Titration Timeline ABG, arterial blood gas; CT, computed tomography; HR, heart rate; BP, blood pressure

Category	Item	Result/Value (Reference Range)
ABG (on presentation) on 1 L/min of nasal oxygen	pH	7.181 (7.35-7.45)
	PaCO₂ (mmHg)	81.6 (35-45)
	PaO₂ (mmHg)	43 (45-65)
	HCO₃⁻ (mmol/L)	30.5 (22-26)
CT imaging before intubation	Lesion characteristics	Enhancing subglottic lesion; length 10 mm in the vertical dimension
	Airway narrowing (%)	~70
Sedated flexible laryngoscopy	Capsule/friability	Thick, nonfriable capsule
	Contact bleeding	Low hemorrhagic risk
Airway management	Extubation	Day 10 from intubation (uneventful)
Propranolol therapy	Timeline monitoring	Start 1 mg/kg/day on day 1 → 2 mg/kg/day on day 4 HR/BP/glucose per institutional protocol; transient hypotension resolved with sedation adjustment

## Discussion

Subglottic hemangioma is a rare but potentially life-threatening cause of upper airway obstruction in neonates and infants [6]. Although most cases progress gradually over weeks [3,4], some may deteriorate rapidly within days and require urgent airway intervention. Misdiagnosis as an upper respiratory infection is common due to overlapping symptoms [4], potentially delaying definitive treatment. In particular, biphasic stridor, absence of positional variation, and rapid progression should prompt suspicion for a fixed subglottic lesion, rather than more common causes such as laryngomalacia. In the present case, careful differential diagnosis and early laryngoscopic evaluation were critical in avoiding diagnostic delay.

Contrast-enhanced CT remains useful for defining the extent of the lesion and degree of airway compromise, but laryngoscopy provides complementary information regarding mucosal integrity and bleeding risk, both of which directly influence airway management. In our patient, repeated laryngoscopy under sedation was helpful in confirming the safety of intubation and avoiding unnecessary tracheostomy. This approach underscores the importance of individualized airway planning in subglottic hemangiomas.

Since the 2008 landmark report of propranolol’s efficacy for infantile hemangiomas [7], this therapy has supplanted corticosteroids and surgical interventions as the first-line treatment, including for subglottic lesions [8]. Multiple studies have demonstrated that, even in severe airway compromise, systemic propranolol can produce rapid improvement within 24-48 hours [4], reducing lesion size and alleviating obstruction. Such early responses may obviate the need for tracheostomy or prolonged intubation in selected patients. In our case, the degree of airway narrowing and hypercapnic respiratory failure raised significant concern for the need for tracheostomy if prolonged airway support became necessary. However, careful airway management, combined with early initiation of propranolol, resulted in marked regression of the lesion and successful extubation by day 10, thereby avoiding tracheostomy altogether. Our experience aligns with previous reports demonstrating that timely propranolol monotherapy can stabilize even severe cases and preserve airway integrity without invasive surgical interventions [4,8].

Close monitoring for adverse effects, such as hypoglycemia, bradycardia, and hypotension, remains essential, especially during initiation and dose escalation of propranolol. Our patient experienced transient hypotension, which resolved promptly with adjustment of sedative agents, emphasizing the need for multidisciplinary coordination. The long-term outcome of subglottic hemangiomas is generally favorable, as most lesions involute with age. However, recurrence or residual airway narrowing may occur, necessitating extended follow-up with laryngoscopic or radiologic surveillance. In our patient, continued propranolol therapy until 14 months of age resulted in sustained remission without recurrence at 24 months. This favorable course supports early initiation and maintenance of propranolol as an effective and safe strategy, even in preterm infants.

## Conclusions

This case illustrates several important clinical lessons. Rapidly progressive biphasic stridor in early infancy should raise suspicion for subglottic hemangioma, and early laryngoscopic evaluation is crucial for diagnosis, risk assessment, and airway planning. Propranolol is highly effective, with the potential for rapid improvement within 24-48 hours, thereby reducing the morbidity associated with tracheostomy or prolonged intubation when initiated promptly, under careful monitoring.
